# Existing capacity to manage pharmaceuticals and related commodities in East Africa: an assessment with specific reference to antiretroviral therapy

**DOI:** 10.1186/1478-4491-7-21

**Published:** 2009-03-09

**Authors:** Paul J Waako, Richard Odoi-adome, Celestino Obua, Erisa Owino, Winnie Tumwikirize, Jasper Ogwal-okeng, Willy W Anokbonggo, Lloyd Matowe, Onesky Aupont

**Affiliations:** 1 Department of Pharmacology and Therapeutics, Makerere University, College of Health Sciences, Box 7072, Kampala, Uganda; 2 Department of Pharmacy, Makerere University, College of Health Sciences, Box 7072, Kampala, Uganda; 3 Management Sciences for Health, Rational Pharmaceutical Management Project, Washington DC, USA; 4 Department of Ambulatory Care and Prevention, Drug Policy Research Group, Harvard Medical School, Boston, Massachusetts, USA

## Abstract

**Background:**

East African countries have in the recent past experienced a tremendous increase in the volume of antiretroviral drugs. Capacity to manage these medicines in the region remains limited. Makerere University, with technical assistance from the USAID supported Rational Pharmaceutical Management Plus (RPM Plus) Program of Management Sciences for Health (MSH) established a network of academic institutions to build capacity for pharmaceutical management in the East African region. The initiative includes institutions from Uganda, Tanzania, Kenya and Rwanda and aims to improve access to safe, effective and quality-assured medicines for the treatment of HIV/AIDS, TB and Malaria through spearheading in-country capacity. The initiative conducted a regional assessment to determine the existing capacity for the management of antiretroviral drugs and related commodities.

**Methods:**

Heads and implementing workers of fifty HIV/AIDS programs and institutions accredited to offer antiretroviral services in Uganda, Kenya, Tanzania and Rwanda were key informants in face-to-face interviews guided by structured questionnaires. The assessment explored categories of health workers involved in the management of ARVs, their knowledge and practices in selection, quantification, distribution and use of ARVs, nature of existing training programs, training preferences and resources for capacity building.

**Results:**

Inadequate human resource capacity including, inability to select, quantify and distribute ARVs and related commodities, and irrational prescribing and dispensing were some of the problems identified. A competence gap existed in all the four countries with a variety of healthcare professionals involved in the supply and distribution of ARVs. Training opportunities and resources for capacity development were limited particularly for workers in remote facilities. On-the-job training and short courses were the preferred modes of training.

**Conclusion:**

There is inadequate capacity for managing medicines and related commodities in East Africa. There is an urgent need for training in aspects of pharmaceutical management to different categories of health workers. Skills building activities that do not take healthcare workers from their places of work are preferred.

## Introduction

Over the past few years, East African countries have experienced a tremendous increase in the volume of antiretroviral drugs. This is a direct result of the commendable global initiatives towards improving access to effective treatment of HIV/AIDS [1,2]. Lack of adequate human resources to support scale-up of treatment programs has been a major constraint to treatment programs. In particular, pharmaceutical supply management systems are notably weak [3], yet they are crucial for successful scale-up of treatment programs [4,5].

To build in-country and regional capacity in pharmaceutical management, Uganda's Makerere University, with technical assistance from the USAID supported Rational Pharmaceutical Management Plus (RPM Plus) Program of Management Sciences for Health (MSH) established the Regional Technical Resource Collaboration (RTRC), a network of academic institutions to build capacity for pharmaceutical management. The initiative [6], which includes institutions from Uganda, Tanzania, Kenya and Rwanda aims to improve access to safe, effective and quality-assured medicines for the treatment of HIV/AIDS, TB and Malaria through spearheading in-country capacity building and operational research activities. To identify specific human resources constraints for pharmaceutical supply management, an assessment was carried out in each of the four countries. The specific objective of the assessment was to determine the existing capacity of the health care system to select, quantify, distribute, and use ARVs; determine the categories of health workers involved in the supply management of ARVs and assess their knowledge and practice with regard to management and use of ARVs; document the nature of current training programs for ART commodities supply management, identify knowledge gaps and suggest necessary intervention to redress the constraints.

## Methods

A cross-sectional survey of fifty governmental and non-governmental institutions accredited to provide ART services in the four countries was conducted in the months of February and March 2005. One researcher from Makerere University and one in-country collaborator carried out the assessment. The assessment used a qualitative research methodology that included interviewing key informants, in-depth interviews of health care workers, and a survey of health facilities and programs.

### Setting and sampling

The survey covered both urban and rural areas and looked at different facets of healthcare provision including, public, private-for-profit, and private not-for-profit sectors. By convenience sampling, a minimum of 10 facilities were targeted in each country and at least three healthcare workers from each facility were interviewed.

### The assessment process

The assessment was standardized across the participating countries through a planning workshop, which brought together collaborators from Makerere University, Harvard Centre for International health, Management Sciences for Health, and two representatives of the AIDS Control Program from each of the four countries. The workshop reviewed the data collection tools, discussed the assessment logistics and process and agreed on time lines. Data collection tools were piloted at three health facilities and two programs in Uganda and their validity and reliability ascertained. These were later excluded from the main study. Permission to carry out the survey was obtained from the national HIV/AIDS control programs of the respective countries. Appointments with heads of the facilities and respondents were made by personnel from the national AIDS control program in each country.

### Interview of key informants

The heads of the National AIDS Control Programs, Ministry of Health Pharmacy Services, and HIV treatment programs were identified as key informants. Using the data collection tool information on; the general features of the country's ARV supply system [accessibility, availability, funding, monitoring and supervision], qualification and training of health care workers involved in supply management of ARVs and related commodities, and the training needs for the supply management of pharmaceuticals was sought in a face to face interview.

### Survey of health facilities and programs

The heads of the facilities providing ART services in the four countries were the key informants at the program level. The survey looked at the types of HIV/AIDS services provided, the existence of guidelines for management of ARVs and related commodities, the qualification of healthcare workers managing the supply of pharmaceuticals, existence of any ongoing skills building activities, and areas covered in ongoing training programs.

### In-depth interviews of health care workers

Structured in-depth interviews of healthcare providers were held with physicians, pharmacists, pharmacy assistants, nurses and clinical officers. Information was sought on training background, knowledge of ARV supply management systems and the quality of the service provided.

### Data analysis

At the end of each survey, questionnaires were checked for completeness, accuracy and consistency. At the end of each assessment, analysis of the data involved discussion with various in-country stakeholders for more in-depth interpretation of perceptions and opinion on possible interventions to address identified problems. Quantitative data were analyzed using EPI INFO Version 3.3, while qualitative data were coded and manually analyzed. No statistical comparisons were made as this was a situational analysis survey without sufficient power for such analysis.

## Results

### ART treatment programs in the four countries

National AIDS Control Programs were responsible for the development and implementation of HIV/AIDS treatment policies in all four countries. Public, private-not-for profit and private-for-profit institutions were involved in HIV/AIDS treatment and care in all the four countries. In the public institutions HIV/AIDS treatment was limited to district level facilities or higher at the time of the assessment.

**Figure 1:**
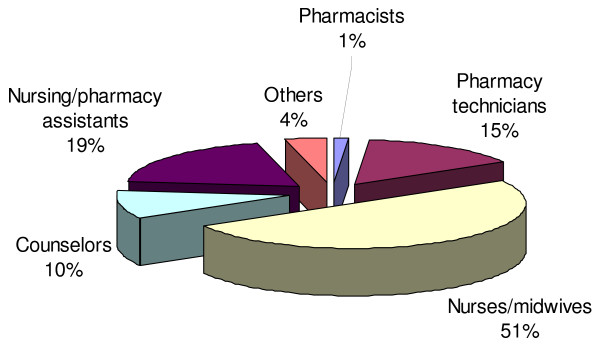
**Distribution of healthcare workers managing HIV/AIDS pharmaceuticals in Uganda**.

A total of 54 facilities involved in ART services were surveyed in the four countries, comprising of 27 public, 18 private not-for-profit, 5 private-for-profit, and 4 academic institutions (Table 1). The majority of facilities were located in urban or peri-urban areas. A total of 110 health workers were interviewed, the majority of whom were pharmacists (32) followed by nurses/midwives (27), doctors (20), pharmacy technicians (20), social workers (9), clinical officer (1) other (1) (Table 2). Generally healthcare workers involved in the pharmaceutical management of ARVs included pharmacists, nurses/midwifes, pharmacy technicians, pharmacy assistants, social workers and administrative staff. In some countries not all categories of health workers were involved in the supply management of ARVs. In Uganda the supply management of HIV/AIDS pharmaceuticals is mainly by lower to mid-level health workers (Figure 1)

**Table 1:** Category of institutions surveyed in the four countries

***Category of Institution***	***Kenya***	***Rwanda***	***Tanzania***	***Uganda***	***Total***
Public Institutions	6	7	8	6	27
Private not-for-profits	5	3	3	7	18
Private for profit	2	1	-	2	5
Academic institutions	-	1	1	2	4

**Total**	13	12	12	17	54

**Table 2:** Healthcare workers interviewed on the supply management of HIV/AIDS pharmaceuticals in Kenya, Rwanda, Tanzania and Uganda

***Profession***	***Kenya***	***Rwanda***	***Tanzania***	***Uganda***	***Total***
Doctors	9	1	2	8	20
Pharmacists	18	6	3	5	32
Nurses/Midwives		8	9	10	27
Pharmacy Technicians	7		2	11	20
Clinical officers				1	1
Social workers		9			9
Others	-	-	1	-	1

Total	34	24	17	35	110

Guidelines for the supply management of HIV/AIDS existed in all the countries but were not always available at treatment centers. Such guidelines however varied from country to country.

### HIV/AIDS Pharmaceutical Management Training

Training on HIV/AIDS mainly focused on the clinical management. Very few programs included pharmaceutical supply management in their training programs. Respondents indicated that there was critical need for training on HIV/AIDS pharmaceutical supply management. Specific areas on HIV/AIDS pharmaceutical supply management where training was needed included the selection of medicines, procurement procedures, quantification of needs, distribution, inventory control/storage and rational prescribing and dispensing. Table 3 shows the areas in HIV/AIDS pharmaceutical management where skills building was required, the perceived cause of the problem and possible interventions. On the preferred modes of delivering training, on-the-job training and short workshops were the preferred by respondents.

**Table 3:** Identified human resource related problems, perceived causes and suggested interventions

**Identified problem**	**Perceived cause**	**Suggested intervention**
Inefficient selection of medicines	• Lack of training on selection methods	• Training on selection
Drug shortages/Expiries	• Inappropriate quantification methods Poor inventory management practices	• Training on quantification methods • Training on inventory management
		
Inappropriate prescribing	• Inadequate training • Insufficient number of prescribers	• Training on appropriate prescribing • Training more prescribers • Review prescribing laws and regulations to allow more health care cadres to prescribe
		
Inappropriate dispensing	• Inadequate training	• Training healthcare workers on appropriate dispensing practices
		
Non-adherence to ART	• Inadequate counseling • Inadequate monitoring and reporting	• Build skills on appropriate counseling techniques • Training on monitoring and reporting
		
Inadequate levels of staffing	• Limited funding for training and education • Poor remuneration and working conditions	• Mobilization of more funding for training and education • Improve remuneration and working conditions
		
**Geographical staffing inequity**	• Preference for working in certain geographical locations such as cities	• Introduce incentives for working in non attractive areas

## Discussion and recommendations

Numerous problems were identified in the four countries with regard to HIV/AIDS pharmaceutical supply management, as was earlier reviewed in sub-Saharan Africa [7]. In this study the major problems pertained to various facets of human resource constraints including inadequate number of personnel being involved in the supply chain, staff being inadequately trained and staff being inadequately remunerated. Lessons from other countries like Thailand and Brazil would come in handy to improve management of ARVs [8]. However local and appropriate interventions are necessary to address the human resource constraints in the supply chain of pharmaceuticals.

The study further showed that few workers had received training on HIV/AIDS pharmaceutical management. The assessment showed a need for training in ARV supply management, and use in the four countries. In addition to training, there is need to develop clear and concise guidelines on the supply management and use of ARVs. Training methods that draw health workers away from their work places for long periods of time are unpopular. On-the-job training and short in-country workshops, with regular follow-up have been reported as effective elsewhere [9].

At the time the assessment was conducted the World Bank and WHO in collaboration with UNAIDS, UNICEF, and the Global Fund for AIDS, Tuberculosis and Malaria (GFATM) were delivering a series of training courses on managing procurement and logistics of HIV/AIDS drugs and related supplies [10]. The target audience, however, were mainly senior staff from governments, donor agencies, international organizations, and NGOs responsibility for the procurement and/or distribution of ARVs and not facility level healthcare workers. The country assessments indicated the need for training with emphasis on health care workers involved with pharmaceutical supply management at the facilities. Collaboration between international organizations with local groups such as academic institutions is likely to produce more sustainable results.

Even though retail pharmacy outlets dispensed ARVs in Kenya, Uganda and Tanzania, clinical training programs on ART seldom included them. Future training initiative should include private pharmacies. In Kenya, policy makers believe that professional bodies such as the Kenya Pharmaceutical Society could contribute significantly to the training of community pharmacists providing ART services.

## Conclusion

Capacity for ARVs supply management in Uganda, Kenya, Tanzania, and Rwanda were found to be limited due many problems. These problems included poor human resource with inadequate skills and capacity to select, quantify and distribute the drugs, with irrational prescribing and dispensing. This calls for There is thus need to provide training in drug supply management in all four countries. Training processes that include local institutions are more sustainable and likely to cover wider geographical areas. The preferred modes of training are on-the-job training and short courses that do not draw participants away from their workplaces.

## Competing interests

The authors declare that they have no competing interests.

## Authors' contributions

PJW: Participated in designing the study, planning, data collection, data analysis and manuscript writing. ROA: Participated in designing the study, planning, data collection, data analysis and manuscript writing. CO: Participated in designing the study, planning, data collection, and manuscript writing. EO: Participated in designing the study, planning and data collection. WT: Participated in designing the study, planning and data collection. JO: Participated in designing the study, planning and data collection. WWA: Participated in designing the study, planning and data collection. LM: Participated in organizing funding, data analysis and manuscript writing. OA: Participated in applying for funding, designing the study, data collection, data analysis and manuscript writing.
